# VR for Situational Awareness in Real-Time Orchard Architecture Assessment

**DOI:** 10.3390/s25216788

**Published:** 2025-11-06

**Authors:** Andrew K. Chesang, Daniel Dooyum Uyeh

**Affiliations:** Department of Biosystems and Agricultural Engineering, Michigan State University, East Lansing, MI 48823, USA

**Keywords:** human–robot interaction, virtual reality, situational-awareness, orchard scouting, teleoperation, point cloud streaming

## Abstract

Teleoperation in agricultural environments requires enhanced situational awareness for effective architectural scouting and decision-making for orchard management applications. The dynamic complexity of orchard structures presents challenges for remote visualization during architectural scouting operations. This study presents an adaptive streaming and rendering pipeline for real-time point cloud visualization in Virtual Reality (VR) teleoperation systems. The proposed method integrates selective streaming that localizes teleoperators within live maps, an efficient point cloud parser for Unity Engine, and an adaptive Level-of-Detail rendering system utilizing dynamically scaled and smoothed polygons. The implementation incorporates pseudo-coloring through LiDAR reflectivity fields to enhance the distinction between materials and geometry. The pipeline was evaluated using datasets containing LiDAR point cloud scans of orchard environments captured during spring and summer seasons, with testing conducted on both standalone and PC-tethered VR configurations. Performance analysis demonstrated improvements of 10.2–19.4% in runtime performance compared to existing methods, with a framerate enhancement of up to 112% achieved through selectively streamed representations. Qualitative assessment confirms the method’s capability to maintain visual continuity at close proximity while preserving the geometric features discernible for architectural scouting operations. The results establish the viability of VR-based teleoperation for precision agriculture applications, while demonstrating the critical relationship between Quality-of-Service parameters and operator Quality of Experience in remote environmental perception.

## 1. Introduction

Smart agriculture integrates connected sensing, data analytics, and automation to enhance food production resilience against labor shortages and climate variability. Networked sensors and satellite imagery enable real-time models that optimize irrigation, nutrient delivery, and pest control, reducing labor requirements per hectare while stabilizing yields under variable environmental conditions [1]. Cloud platforms enable remote farm management with a single technician across multiple sites, while variable-rate implements and decision-support algorithms minimize input waste and emissions, aligning with emerging policy incentives for agricultural sustainability. Within this digital ecosystem, robotics represents the technological evolution of traditional mechanization. Autonomous and semi-autonomous field machines now perform commercial-scale operations, including strawberry harvesting, apple thinning, row crop weeding, and targeted spraying, achieving 40–90% pesticide reduction while approaching 50% of the picking efficiency of skilled labor in specialty crops (Bac et al., 2014; Oberti et al.) [2,3]. Commercial platforms such as Naïo Technologies’ https://www.naio-technologies.com/en/home (accessed on 30 October 2025) Oz and Ted weeding robots in European vineyards, John Deere’s See & Spray https://www.deere.com/en/sprayers/see-spray-ultimate (accessed on 30 October 2025) precision herbicide systems, and FFRobotic’s https://www.ffrobotics.com (accessed on 30 October 2025) apple-harvesting platforms demonstrate the advancing capabilities of perception-guided manipulators and autonomous rovers. However, full autonomy remains constrained by unstructured terrain, dense and repetitive foliage, variable ambient lighting, limited onboard computational resources, and safety requirements for human–robot coexistence [4]. These limitations necessitate supervised autonomy approaches, where Human-in-the-Loop (HITL) systems preserve operator oversight for critical decisions while leveraging robotic precision and endurance [5]. HITL architectures enable humans to apply domain knowledge and diagnostic capabilities to address knowledge gaps in automated systems, particularly in dynamic and unstructured agricultural environments, where cognitive awareness, perception, and intuition remain inherently human capabilities that complement automated processes [6,7]. Research indicates that human–machine collaboration in agricultural operations can outperform either component operating independently, while reducing operational costs and completion times compared to traditional methods [6]. However, challenges remain in developing viable HITL systems, particularly in implementing teleoperation interfaces that provide sufficient situational awareness while accommodating the constraints of agricultural environments.

Existing teleoperation systems have demonstrated their feasibility across multiple agricultural contexts, utilizing conventional interface paradigms. Peña et al. [8] developed a teleoperated anthropomorphic robot for tending small urban crops in structured environments, employing a motorized rail system that extends workspace coverage while utilizing environmental sensors (temperature, humidity, light) and cameras for feedback to enable tasks including sowing, irrigation, pruning, and fertilization through a 5-DOF manipulator with multifunctional end-effector capabilities. Ref. [9] addressed operator skill limitations by introducing a tablet-based game-like interface that enables support operators to assist novice tractor drivers during challenging maneuvers, providing real-time feedback through onboard sensors, including position, orientation, and camera imagery for intuitive remote guidance and mode switching between manual and autonomous operation. Similarly, ref. [10] proposed a shared control framework for table-grape harvesting robots that dynamically switches between autonomous, teleoperation, and hand-guiding modes to improve efficiency and adaptability in complex agricultural environments, while ref. [5] evaluated various human–robot interaction modes for teleoperating semi-autonomous sprayers in vineyard environments, demonstrating that multiple camera views combined with PC-based keyboard interfaces significantly improved task efficiency and user satisfaction compared to single-view head-mounted displays or gamepad controllers.

Recent advances have explored immersive technologies to address the limitations in situational awareness inherent in traditional teleoperation interfaces. Ref. [11] introduced the VitRob Pipeline, combining virtual reality with robotics for precision agriculture fruit harvesting through the integration of ROS, Unity, and AI-based 6DOF pose estimation, achieving sub-second latency for real-time control and visualization of ground vehicles and robotic manipulators. Ref. [12] proposed an immersive teleoperation framework utilizing UAV-captured footage reconstructed through Structure-from-Motion Multi-View Stereo (SfM-MVS) techniques to generate static 3D maps for visualization on a standalone Virtual Reality Headset during live UAV control; however, it still acknowledged limitations in capturing environmental dynamics. Ref. [13] demonstrated VR system feasibility for tomato harvesting and leaf inspection tasks, achieving 90% robot–plant interaction success rates using RGB imagery rendered in virtual environments from robot perspectives, while subsequent work [14] on strawberry identification and classification highlighted occlusion-related limitations when relying on single-camera feedback systems. Ref. [15] developed a real-time 3D reconstruction and immersive teleoperation system combining VR technology with Microsoft Kinect RGB-D sensors to create realistic 3D models of unstructured agricultural environments streamed to a tethered VR headset for enhanced first-person perspective control. The reviewed literature demonstrates teleoperation applications predominantly concentrated in harvesting operations (fruit picking, grape harvesting), spraying and pesticide application, basic cultivation tasks (sowing, irrigation, pruning), and vehicle navigation assistance. However, critical agricultural operations that rely on 3D spatial information of the environment, such as scouting for architectural traits that inform orchard management decisions, remain largely unexplored in teleoperation contexts. These scouting operations require a detailed spatial understanding of three-dimensional plant structures, which current teleoperation systems inadequately address.

Furthermore, existing feedback technologies predominantly rely on a 2-dimensional perspective, which provides limited spatial context to operators. Additionally, while VR-based systems demonstrate improved immersion and spatial awareness compared to conventional interfaces, current approaches primarily visualize robot status, providing a planar view of the agricultural environment, similar to 2D frames from the captured sensors. Refs. [10,13] take an extra step by visualizing the environment in 3D to provide a more immersive and richer spatial understanding of the environment; however, the former’s implementation is limited to static environments, while the latter, though real-time, was only demonstrated on a tethered setup and may hinder freedom of motion and, even more, adoption of the technology. To address these limitations, this work presents a pipeline for providing a dynamic spatial representation of the environment as feedback to teleoperators through virtual reality. The pipeline tackles bandwidth constraints inherent in agricultural teleoperation through a selective streaming module that transmits spatial data based on operator localization within reconstructed environments. The stream is then parsed in Unity and rendered through an adaptive point cloud shader system for real-time visualization performance. The system’s capabilities are demonstrated through scouting operations in high-density apple orchards, where three-dimensional architectural representations enable the assessment of canopy density, branch structure, and growth patterns that are critical for informed orchard management decisions. This paper presents the pipeline architecture and core algorithms (Section 2), describes the experimental implementation, including equipment specifications and study site characteristics (Section 3), evaluates system performance and demonstrates practical applications (Section 4), and concludes with a discussion of the findings and future research directions (Section 5).

## 2. Methodology

### 2.1. Overview

**Figure 1:**
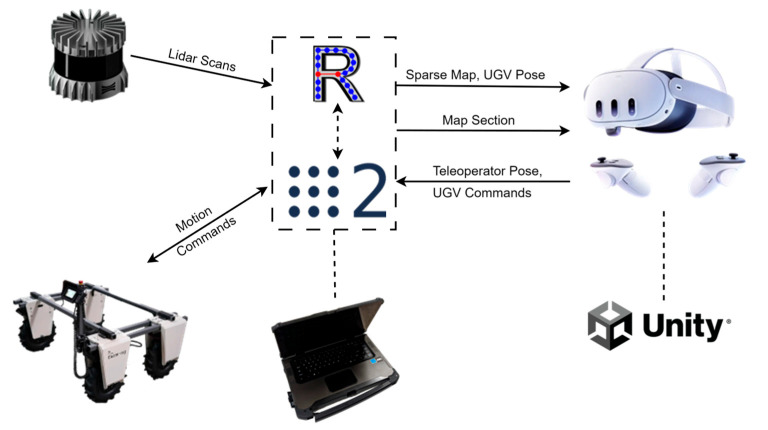
Overall pipeline for realizing Visual Feedback based on Dynamic Point Clouds in a Virtual-Reality-Based Teleoperation workstation.

Our proposed methodology presents a complete pipeline for streaming and rendering point clouds to provide visual feedback during teleoperation in agricultural scouting applications, see Figure 1. The system integrates ROS2 middleware with Unity’s runtime framework, bridging communication between an AMIGA robot, a robotics platform developed by Farm-ng Inc. (Watsonville, CA, USA) [16], and the teleoperator workstation, see Figure 1. The pipeline addresses two fundamental challenges in remote orchard inspection: efficient data transmission and adaptive visualization. Dense 3D mapping is performed using RTABMap with connected vision sensors to capture high-fidelity orchard environments, suitable for analyzing architectural traits and creating a dense map [17,18]. To manage communication constraints, the system generates sparse map representations through downsampling of the dense reconstructions.

The pipeline comprises three core modules that work in tandem to optimize teleoperator situational awareness. First, selective streaming (Section 2.2) implements view-aware data transmission that reduces bandwidth requirements while maintaining spatial context through on-demand high-resolution region requests. Second, high-performance binary parsing (Section 2.3) converts ROS2 PointCloud2 messages into Unity-compatible GPU data structures for subsequent shader-based rendering. Third, adaptive rendering (Section 2.4) employs distance-based Level of Detail (LOD) scaling within Unity’s Universal Render Pipeline, where each point is rendered as a dynamically sized disk that balances visual fidelity with rendering performance based on the teleoperator’s virtual position. This integrated approach enables real-time remote scouting capabilities while addressing the competing demands of network efficiency, system performance, and visual quality inherent in agricultural teleoperation scenarios.

### 2.2. Selective Streaming

As the robotic platform traverses the orchard environment, continuous sensor data capture results in a dynamic map with exponentially growing density and size. While dense point clouds provide high-fidelity representations crucial for accurate scene understanding, transmitting complete datasets is limited by bandwidth limitations and network latency constraints [4]. These communication bottlenecks manifest as dropped packets and degraded real-time performance, directly impacting visual feedback. To address these limitations, we implement a two-tier streaming approach that balances visual fidelity with communication efficiency. The primary stream consists of a sparse representation generated by uniformly downsampling the dense point cloud by a factor β, providing continuous low-latency updates to maintain spatial awareness. This downsampled representation serves as the baseline visualization rendered at the teleoperator workstation, as detailed in Section 2.3.

The selective streaming mechanism enhances this baseline through an on-demand, view-aware capability that leverages the teleoperator’s spatial context within the mapped environment. Real-time localization tracks the teleoperator’s 6-DOF pose within the orchard map, enabling precise determination of their viewpoint and orientation. When operators identify regions requiring higher detail, they can request enhanced data for specific map sections, through an interactive request-response protocol. These high-resolution requests utilize the teleoperator’s head-mounted display (HMD) field-of-view (FOV), $\theta_{FOV}$ parameters combined with their localized pose $T_{op}$ to construct a view frustum.

(1) $$P_{visible}\;=\;\{p\;\in \;P_{dense}\;:\;p\;\in \;F\left(T_{op},\;\theta_{FOV}\right)\;\}$$

(2) $$F\left(T_{op},\theta_{FOV}\right)=\{p\in R^{3}:\;\left\{\begin{array}{c} \left(p-t_{op}\right)\cdot f>0\\ |\;(p-t_{op})\cdot r\;|\leq (p-t_{op})\cdot f\cdot \tan\left(\theta_{FOV}/2\right))\;\\ \left|\;(p-t_{op})\cdot u\;|\leq (p-t_{op})\cdot f\cdot \tan\left(\theta_{FOV}/2\right)\right) \end{array}\right.$$

where

- $P_{visible}$ = culled point set transmitted to teleoperator;
- $P_{dense}$ = complete dense point cloud;
- $R_{op}$ = teleoperator’s orientation;
- $t_{op}$ = teleoperator’s position;
- $T_{op}=\left[R_{op}|t_{op}\right]$ = teleoperator’s 6-DOF pose transformation;
- f,r,u = forward, right, up vectors from $R_{op}$;
- $\theta_{FOV}$ = HMD field of view angle.
- *p* = an arbitrary point in 3D space

**Figure 2:**
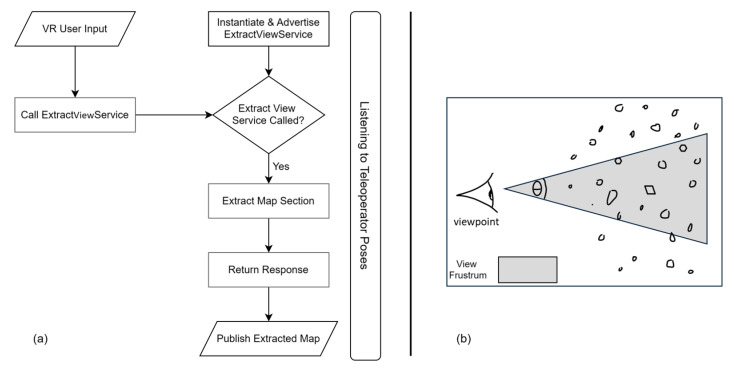
(**a**) Sequential Flow of the Selective Streaming Process. (**b**) Illustration of view frustrum culling.

The frustum acts as a spatial filter on the dense point cloud, culling data outside the operator’s immediate viewing area while preserving high-fidelity information within their visual attention zone, see Figure 2. The resulting view-aware implementation reduces bandwidth requirements by transmitting only visually relevant data, effectively implementing view-frustum culling at the network level rather than solely at the rendering stage. This selective approach aims to improve teleoperator Quality of Experience (QoE) by providing adaptive detail-on-demand while maintaining real-time system responsiveness through efficient bandwidth utilization.

### 2.3. PointCloud Parsing

Unity’s native rendering pipeline lacks built-in support for parsing Robot Operating System 2 (ROS2) PointCloud2 [19] message formats, requiring custom binary data parsing for point cloud data visualization. The parsing module was implemented using unsafe C# pointer arithmetic operations with fixed memory pinning to prevent garbage collection interference during data extraction. Spatial coordinates ‘x’, ‘y’, and ‘z’ and the packed BGRA ‘rgb’ fields are extracted from byte streams through pointer dereferencing operations. The received point cloud’s coordinate system undergoes transformations from ROS2’s right-handed (Z-up, X-forward, Y-left) convention to Unity’s left-handed (Y-up, Z-forward, X-right) convention, following the mapping:

$$P_{Unity}=\left(-y_{ROS2},z_{ROS2},x_{ROS2}\right),$$

where $P_{Unity}$ denotes the transformed position in Unity space and ($x_{ROS2}$,$\;y_{ROS2}$,${\;z}_{ROS2}$) represents the original point coordinates from ROS2, while RGB values were repacked into Unity-compatible RGBA format through bit-shifting operations. The parsed data are then consolidated into a compact 16-byte Point Data struct containing Vector3 positions and packed 4-byte unsigned integer color values. To ensure real-time performance is maintained, explicit message dropping **is** implemented via thread-safe locking mechanisms, where incoming messages are discarded when parsing operations exceed sensor data rates. The resulting structured point data are then transferred to GPU Compute Buffers for subsequent shader-based rendering operations.

### 2.4. Adaptive Rendering

While selective streaming addresses bandwidth constraints, the received sparse point clouds present their own visualization challenges. Sparse representations inherently lack visual fidelity, which affects depth perception and spatial understanding—critical for teleoperation tasks. Distance is a critical factor in perception quality—points appear increasingly disconnected and difficult to interpret at closer distances. Conversely, rendering very dense point clouds while providing high visual fidelity in real-time can incur high computational costs, resulting in poor rendering performance due to pixel overdraw and memory bandwidth saturation, which ultimately degrades teleoperator QoE through reduced frame rates and system responsiveness.

At the core of the adaptive rendering module is a geometry shader program that borrows from an open-sourced Point Cloud renderer implementation [20], which uses disk representations for individual points. The rendering module begins with a vertex shader that receives vertex IDs to index into a GPU ComputeBuffer containing packed point position and color data, unpacks RGB color components using bit manipulation, and performs spatial transformations to represent points within the camera’s coordinate system and view frustum. Subsequently, the geometry shader calculates a distance-scaled radius *R*(*P*) that determines the final size of each point disk based on the displacement magnitude from camera position *C* to point *P*, bounded by predefined distance *d* and scale *α* parameters:

(3) $$R\left(P\right)=s_{0}\cdot k\cdot \mathrm{lerp}\left(\alpha_{\max},\alpha_{\min},\frac{\mathrm{clamp}\left(||P-C||,d_{\min},d_{max}\right)-d_{\min}}{d_{\max}-d_{\min}}\right)$$

where

- *s*_0_ = base point size;
- *k* = global scale factor;
- *||P − C||* = displacement magnitude from camera *C* to point *P*;
- *α_max_*, *α_min_* = max/min scale factors;
- *d_min_*, *d_max_* = distance bounds.

The calculated radius R(*P*) is then projected to screen space using the projection matrix elements, and the shader determines the optimal number of vertices required to balance visual quality with performance. This process generates vertices that form a polygonal disk $D\left(P\right)$ representative of each point according to the following formula:

(4) $$D\left(P\right)=\left\{P+\left|P_{11}P_{22}\right|\cdot R\left(P\right)\cdot \left(\cos\left(\frac{\pi i}{N\left(R\left(P\right)\right)}\right),\sin\left(\frac{\pi i}{N\left(R\left(P\right)\right)}\right)\right)\;|\;i\in \left[0,\;2N\left(R\left(P\right)\right)-1\right]\right\}$$

(5) $$N\left(R\left(P\right)\right)=\min\left(\frac{P_{22}\cdot RP\cdot h}{5\cdot P.w+1},\;4+2\right)$$

where

- *P* = point position in clip space;
- *P*_11_ = horizontal projection matrix element (UNITY_MATRIX_P._11);
- *P*_22_ = vertical projection matrix element (UNITY_MATRIX_P._22);
- *R(P)* = radius function from previous equation;
- *h* = screen height in pixels;
- *P.w* = homogeneous coordinate (depth) of point P;
- *I* = vertex index iterator.

The generated disk polygons are then rasterized into individual pixel fragments, where the fragment shader applies color through interpolation of vertex color attributes, completing the adaptive point cloud rendering process. While both our implementation and [16] use disc-shaped polygons with geometries for representation, the latter is restricted to the Built-In Render Pipeline, relies on point cloud files from the filesystem, and lacks adaptiveness in scaling of the disc representations.

## 3. Experimental Setup

### 3.1. Study Area and Equipment

**Figure 3:**
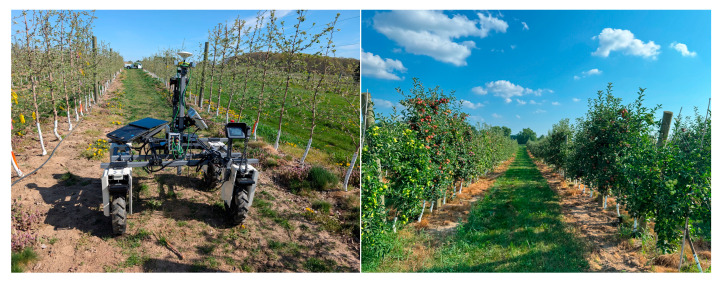
(**Left**): The UGV with the data collection unit in the orchard in spring. (**Right**): A row of the orchard during the summer.

Data collection was conducted at the Plant Pathology Research Center in East Lansing, Michigan, within a 240 × 350-foot high-density orchard block, see Figure 3. The orchard consists of four apple cultivars (Rubymac, Buckeye Gala, Royal Red Honeycrisp, and Gold Rush) arranged in rows spaced 15 feet apart, with trees positioned 3 feet apart within rows. The experimental platform utilized an Amiga UGV (Farm-ng Amiga, Watsonville, CA, USA) equipped with an Ouster OS0-128 LiDAR (Ouster, Inc., San Francisco, CA, USA) sensor. Two datasets from the Orchard were used for evaluation. These were collected from the first and second row-alleys of the eastern end orchard during the summer and spring of 2025, respectively. The data collection unit’s configuration for the spring dataset was: a lidar mode of 1024 × 10, with the minimum and maximum range of the lidar projection set to the default 0 and 1000 m, respectively, and 360-degree coverage for the azimuth. The UGV traversed the row-alleys at a speed of 100 ft/min. For the summer dataset, the lidar mode was set to 4096 × 5 with the min and max ranges set to 1.2 and 6.0 m. The azimuth was set to a start of 30 degrees and an end of 330 degrees based on the lidar coordinate frame [21]. Both datasets were recorded on the filesystem of a field onboard the UGV, which interfaced with the Lidar via a Gigabit connection, as ROS2 Bags.

### 3.2. Dynamic Map Reconstruction

The LiDAR sensor generates PointCloud2 messages containing spatial coordinates (x, y, z), reflectivity, signal strength, ring, range, and near-infrared intensity for each measured point. The absence of RGB color information in the raw sensor data presents limitations for operator perception during teleoperation tasks. To address this constraint, the 16-bit reflectivity field was utilized as a basis for pseudo-color visualization. Data preprocessing involves filtering out invalid measurements by retaining points with finite spatial coordinates and reflectivity values that exceed zero. Reflectivity normalization is performed using the 1st and 99th percentiles of the distribution as minimum and maximum bounds, respectively, thereby mitigating the influence of extreme outliers. To optimize computational performance, these percentile values are cached and updated every 20 scan cycles. The normalized reflectivity values are subsequently mapped to the gist_earth [22] colormap, which exhibits favorable characteristics for topographical data visualization [23]. The resulting RGB values are encoded in 32-bit format and integrated into the point cloud structure alongside the spatial coordinates before publication.

The enhanced point cloud messages serve as input to RTAB-Map’s ICP odometry node, which performs scan-to-scan registration using Point-to-Plane Iterative Closest Point (ICP) algorithms to estimate robot pose and generate odometry data. This odometry establishes a reference coordinate frame that enables computation of spatial transformations between the robot’s current pose and the global map coordinate system. The downstream SLAM module integrates this odometry information with incoming LiDAR measurements, executing pose-graph optimization through ICP-based registration between mapping keyframes. Through incremental graph construction and optimization, the system combines odometry constraints with scan alignments to generate a spatially consistent and dense three-dimensional representation of the orchard environment.

### 3.3. Virtual Reality Application

A Unity-based virtual reality application was developed, targeting the Meta Quest 3 headset, utilizing the Vulkan graphics API and Universal Render Pipeline (URP) for rendering performance, as well as the Multipass Stereo Rendering mode. The application architecture utilized the XR Interaction Toolkit to establish the teleoperator interface framework, while the ROS-TCP-Connector facilitated bidirectional communication with the ROS2 server. The virtual environment features a three-dimensional surrogate representation of the UGV, providing spatial context and enabling potential remote-control functionality. Bidirectional pose tracking was implemented to maintain spatial coherence between the physical and virtual environments. The teleoperator’s head pose within the VR environment is transmitted to the ROS2 backend as a *PoseStamped* message, with the frame identifier corresponding to the reference coordinate frame established in Section 2.2, thereby localizing the operator’s viewpoint within the map coordinate system. Conversely, the robot’s spatial transforms within the map are published as ROS2 topics and consumed by the VR application to update the virtual robot model’s position and orientation in real-time.

Coordinate system alignment between Unity’s Right-Up-Forward (RUF) convention and ROS2’s Forward-Left-Up (FLU) standard for teleoperator and robot pose data was handled through the ROS2-TCP-Connector’s extended message type conversion utilities, while incoming streamed point clouds were processed using the PointCloud parser detailed in Section 2.3. The teleoperator interface offers switching capabilities between First Person View (FPV) and Fly Camera perspectives, along with toggleable remote-control functionality. However, the latter was not utilized in the current experimental setup. A service client for dynamic map extraction was integrated into the application runtime, enabling real-time queries based on the teleoperator’s field of view. The service invocation conveys the operator’s viewpoint field-of-view along with an associated coordinate frame identifier. Upon execution, the service returns a Boolean flag indicating the success or failure of the request, accompanied by a textual message providing contextual information regarding the outcome.

### 3.4. Evaluation Protocol

The proposed pipeline was evaluated using recorded datasets from Section 3.1, stored as ROS2 bag files and processed through streaming modules on an onboard computer equipped with an Intel i7-1370P CPU, 32 GB system memory, and Ubuntu 22.04.5 LTS (64-bit). VR application testing was conducted using two configurations: a standalone Meta Quest 3 headset operating at a 72 Hz display refresh rate, and a PC-tethered setup utilizing a Ryzen 7 5800H processor, RTX 3060 mobile graphics card with 6 GB memory, 32 GB system memory, and Windows 11 operating system connected to the headset via Air Link. The Unity Editor was configured to use DirectX 11 as the graphics API, and the headset refresh rate was adjusted to 120 Hz for the PC-tethered configuration. Performance comparisons of the proposed methods (Section 2.3 and Section 2.4) involving streaming and rendering of the complete published map from RTABMap were conducted against Methods 1 and 3 from [24] with the parameters shown in Table 1. Method 1 was selected based on its performance characteristics among available URP-compatible approaches, while Method 3 was chosen for its use of primitive representations and computing buffers that parallel the proposed approach in this work. For brevity, Hydran00’s Method 1 and Method 3 are hereafter referred to as Hydran001 and Hydran003, respectively. Each VR application test session required the teleoperator to follow the recorded UGV trajectory while maintaining orientation toward the trajectory origin until the UGV reached its endpoint, followed by a return fly-through toward the map’s origin.

A separate evaluation session was conducted, following the same protocol, to assess the method described in Section 2.2. For this evaluation, the base point size was set to 0.015 m for downsampled point clouds, while dense point clouds maintained a point size of 0.01 m. The uniform sampling parameter β was set to 4. Throughout each evaluation session, performance metrics, including the number of streamed points, received point cloud messages, processed point cloud messages, application framerate, and frame processing times, were logged at one-second intervals to establish performance baselines for comparative analysis. All testing computation hardware devices—the standalone headset, onboard computer, and PC—were connected to a WiFi 6 LAN.

## 4. Results and Discussion

The evaluation protocol revealed varying performance characteristics across different hardware configurations and methods. The proposed method and Hydran003 were executed successfully on Meta Quest 3 for both datasets. However, Hydran003 consistently crashed before completing the full evaluation protocol, preventing the return fly-through to the map origin from being completed. Hydran001 operated on the headset with UGV odometry data reflected correctly in the surrogate transformations, but failed to display point clouds. Consequently, the evaluation of the summer dataset was conducted on a PC. Comparative analysis between methods was performed using identical hardware configurations and datasets for each comparison. Performance analysis was conducted on the logged data, with results presented in Table 2 and Table 3 for runtimes based on Standalone VR Headset and Table 4 for runtimes based on the Unity Editor.

The Ouster-128 3D LiDAR system employed in this study generates 128 channels of point data across defined azimuth blocks, establishing horizontal spatial resolution parameters for environmental scanning.

**Figure 4:**
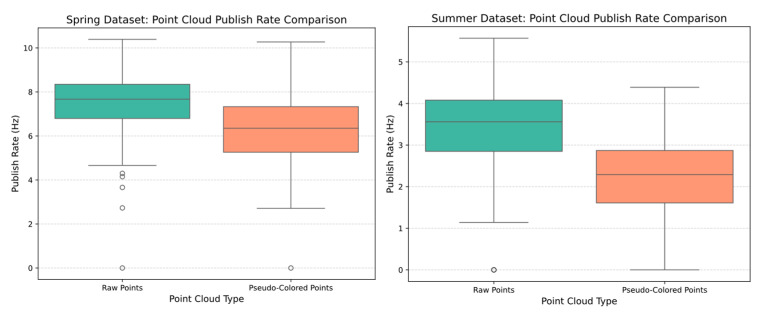
Comparison of the rate at which raw point clouds and their corresponding pseudo-colored versions are published across both datasets.

While the sensor output lacks inherent RGB color information, it provides reflectivity measurements representing the intensity of reflected laser pulses. This reflectivity data serves as a material property indicator, enabling discrimination between objects of similar geometric profiles but different surface characteristics, such as distinguishing trellis wire from thin organic twigs. The implementation of pseudo-coloring through the gist_earth colormap transforms reflectivity values into visual discriminators, enhancing geometric feature identification compared to monochromatic point cloud representations. The pseudo-coloring enhancement introduces computational overhead that reduces publish rates for colored point cloud topics, with performance degradation correlating to point cloud density. Comparative analysis reveals that the summer dataset exhibited 38.11% slower average publishing rates compared to the spring dataset’s 16.18%, see Figure 4. These performance variations demonstrate the trade-off between visual enhancement and real-time processing requirements.

Individual LiDAR scans provide temporal snapshots with limited utility for comprehensive environmental mapping. The integration of RTABMap and Iterative Closest Point (ICP) algorithms for odometry and mapping registration enables the generation of persistent maps that preserve pseudo-color information. The resulting map data, published as a PointCloud2 message, contains coordinate fields (x, y, z) and RGB color information as 32-bit floats with respective offsets of 0, 4, 8, and 16 bytes. This data structure yields a point step of 32 bytes per point, utilizing only 16 bytes of meaningful information. The sparse map representation maintains identical field structure but achieves a reduced point step of 20 bytes through uniform downsampling, incorporating 4-byte padding between ‘z’ and ‘rgb’ fields to preserve consistent field access patterns.

**Figure 5:**
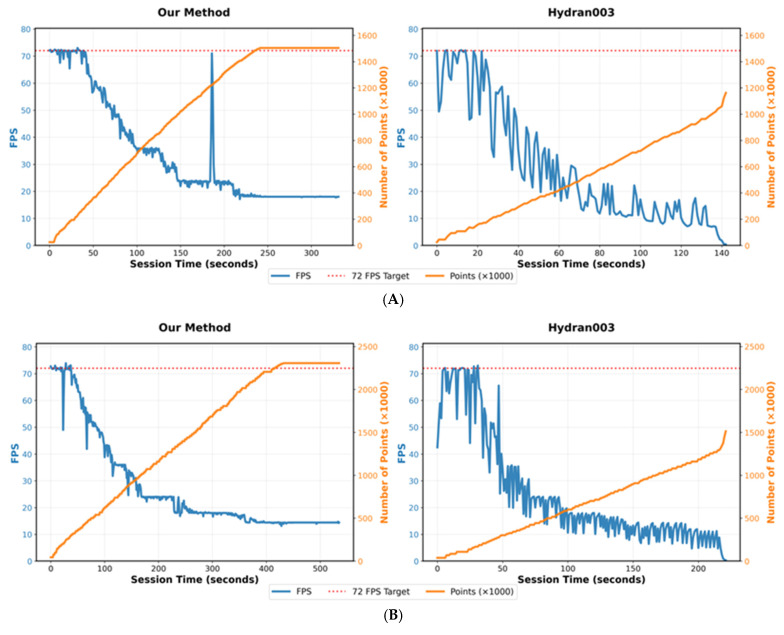

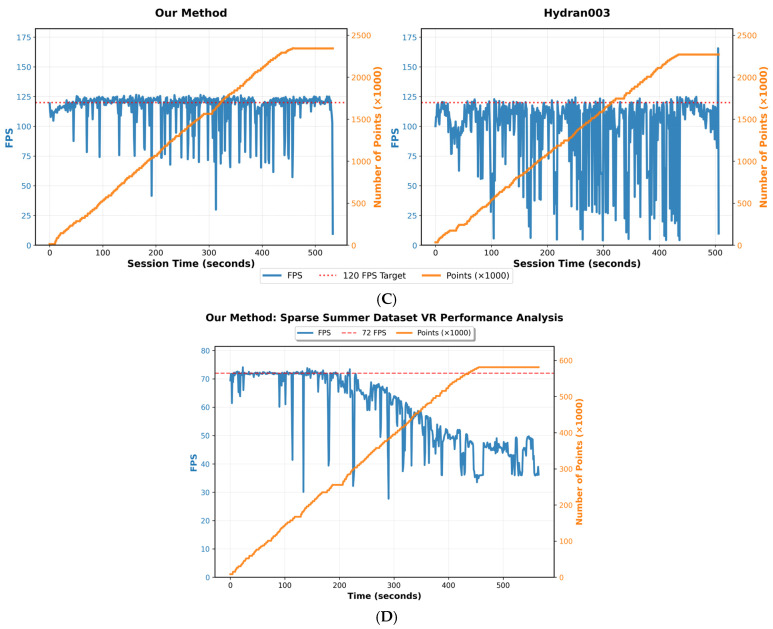
VR Runtime Performance: FPS and Rendered Points against Elapsed Time comparisons on Meta Quest 3 @ 72FPS for (**A**) spring dataset; (**B**) summer dataset; (**D**) downsampled point clouds (**C**) summer dataset on PC @ 120FPS comparisons.

Parsing of received point cloud data presents bottlenecks for large datasets, since each point must be processed sequentially. This aspect is particularly relevant for RTABMap outputs, as RTABMap provides the most recent version of the entire map, rather than only recent segments, thereby increasing the data volume. While computational effort scales linearly with point count across all parsing approaches, efficiency depends on internal operations during parsing. Methods that minimize function calls and processing overhead, such as fixing the message’s byte array in memory and using arithmetic pointers, offer lower overhead compared to alternatives. As a result, this approach delivers a smoother experience with fewer frame drops, which were qualitatively experienced as jitters and quantitatively captured across all comparative runs, with PC-based comparisons appearing more pronounced. The shaders’ LOD system prioritizes closer points, reducing overall resource consumption and enabling the system to achieve target framerates, thereby improving performance. Runtime performance throughout the evaluation sessions and across three side-by-side comparisons against the proposed method showed better performance by 10.2–19.4%. The fly-through experience in the evaluation protocol, which occurred during application execution, isolated performance impacts due to parsing operations when no new messages required parsing. This effect was observed in PC-based evaluations, where the proposed method demonstrated a 5.9% performance advantage, see Figure 5.

Qualitative assessment of visual perception revealed distinct differences between methods across varying distances in the virtual environment. Both Hydran00 methods exhibited improved visual continuity at greater distances from objects, but displayed discontinuous point compositions when positioned closer to mapped features. This discontinuity was more apparent with Hydran001, which used point vertices exclusively for visualization, and less pronounced with Hydran003 due to its scalable point sizing. This phenomenon of varied visual perception across distances is constrained by the human eye’s finite angular resolution. At greater distances, angular separation between points decreases below the threshold of human visual discrimination, rendering them imperceptible [25] and creating the perception of continuous point sets. Discontinuity, which remains discernible at close range, conveys less accurate representations of mapped objects. This sparse nature compromises immersion, and for architectural scouting operations, decision-making processes that depend on affordance derived from immersion are degraded [26].

**Figure 6:**
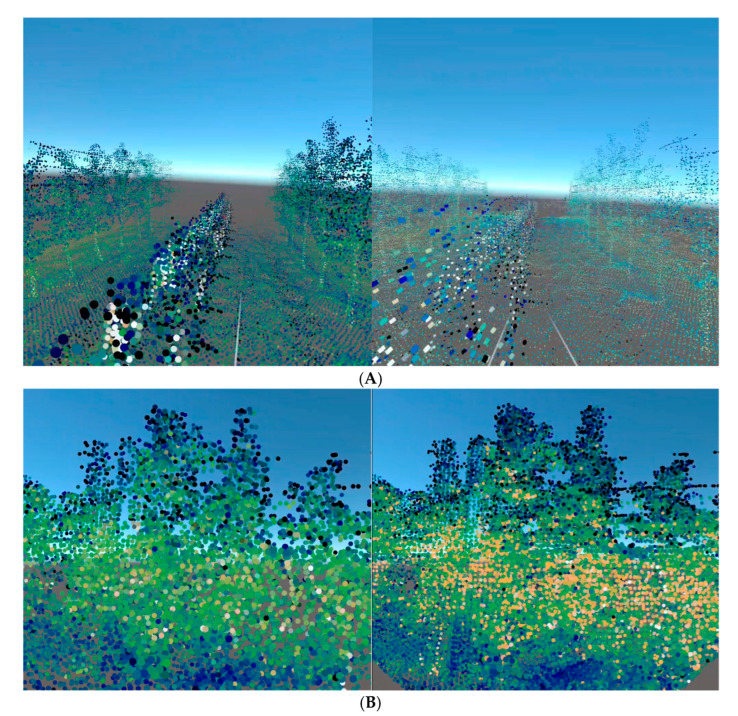
Qualitative Comparisons (**A**) Our Methods (**left**) rendering quality vs. Hydran003 (**right**) on the spring dataset. (**B**) Sparse Representation of an orchard section (**left**) vs. a dense extracted version of the same section.

The proposed method demonstrated opposite rendering characteristics, with points beyond the Upper Distance Bound (d_max_) appearing invisible due to imperceptibly small sizes, while closer objects appeared more continuous through dynamically scaled point sizes that created spatial overlaps at proximity. The LOD implementation of dynamically scalable primitive shapes to model points mitigates effects posed by sparse point clouds, enabling qualitative discernment of tree architecture. This approach made even the downsampled map’s geometric features discernible compared to Hydran001 and Hydran003. The reduced point density of the downsampled point clouds enabled a 112% increase in mean framerate over the adaptive method using the complete map, see Figure 6.

Effective HITL operations depend on both Quality of Service (QoS) and Quality of Experience (QoE) parameters to achieve responsive performance for both machine and human components. QoS encompasses measurable system parameters, such as latency, frame rate, and processing throughput, while QoE encompasses human factors, including visual perception and immersion, that shape operator interaction with teleoperation systems. The connection between these quality parameters and situational awareness lies in their impact on information processing capabilities during orchard scouting operations. Situational awareness operates through three hierarchical levels: perception of environmental elements, comprehension of the current situation, and projection of future status [27]. Since perception forms the foundational tier, any degradation in QoS parameters that compromises visual continuity and framerate stability will undermine an operator’s ability to perceive remote orchard environments accurately. The experimental findings demonstrate this relationship through the performance trade-offs observed between parsing efficiency, rendering fidelity, and real-time responsiveness. The proposed method’s dynamic point scaling and LOD implementation address this challenge by maintaining visual continuity at close range while preserving computational efficiency, thereby supporting the perceptual foundation necessary for architectural scouting decisions. The 112% framerate improvement achieved through downsampled representations, combined with the preservation of geometric feature discernibility, illustrates the practical balance required between QoS optimization and QoE preservation in VR teleoperation contexts.

## 5. Conclusions

This work demonstrates the viability of Virtual Reality as a platform for teleoperation in orchard scouting applications. The proposed method demonstrated substantial performance improvements and enhanced visualization capabilities compared to existing approaches, with framerate enhancements ranging from 10.2% to 19.4% in direct comparisons and up to 112% when utilizing downsampled representations. Qualitative assessment confirmed the method’s ability to discern various tree geometries and support structures such as trellis lines within orchard environments, supporting architectural decision-making processes required for tree training systems and pruning operations. The integration of pseudo-coloring through reflectivity data and dynamic point scaling addressed the fundamental challenge of maintaining visual continuity across varying distances, directly supporting the perceptual foundation of situational awareness. The implementation of LOD successfully balanced computational efficiency with geometric feature preservation, enabling real-time performance on both standalone and PC-tethered VR configurations. Several limitations warrant consideration for future development. RTABMap’s periodic updates of the complete map can overwrite distinct points, creating spatial coherence loss across temporal scales, particularly in cases of self-occlusion during foliated seasons. The computational expense of whole-map updates presents challenges for resource-constrained real-world deployments. Additionally, the standalone implementation’s reliance on the multi-pass stereo-rendering mode introduces performance overhead compared to single-pass instanced rendering, which lacks out-of-the-box support in URP geometry shaders. However, this work did not address networking constraints that could impact real-world teleoperation scenarios, representing a critical area for future investigation. The qualitative evaluation of 3D scenes through 2D projections cannot fully represent the immersive experience within the virtual environment, necessitating more rigorous assessment methodologies. Future work will address these limitations through improvements spanning map creation processes to rendering modalities on standalone headsets, while incorporating networking-related QoS parameters to achieve a comprehensive evaluation of the teleoperation system. Additionally, a more comprehensive qualitative analysis is needed.

## Figures and Tables

**Table 1:** Parameters for the Point Cloud Renderers. The same base point size was also adopted from Hydran003.

Parameter	Value
Base Point Size, s_0_ (m)	0.01
Global Scale Factor, *k*	1
Maximum Scale Factor $\alpha_{max}$	2
Minimum Scale Factor $\alpha_{min}$	0.01
Upper Distance Bound d_max_ (m)	12
Lower Distance Bound d_min_ (m)	0.1

**Table 2:** Performance comparison of the VR application running on a standalone VR Headset across the Spring and Summer Datasets.

Method	Metric	Spring Dataset	Summer Dataset
Ours	Session Duration (s)	338.1	549.6
	Mean FPS	33.91 ± 18.96	27.76 ± 18.03
	Coefficient of Variation	0.559	0.351
	25th–75th Percentile (FPS)	18.0–46.5	14.4–35.5
Hydran00 Method 3	Session Duration (s)	143	281.1
	Mean FPS	28.39 ± 21.52	25.18 ± 20.51
	Coefficient of Variation	0.758	0.814
	25th–75th Percentile (FPS)	11.2–42.8	11.7–32.5

**Table 3:** Performance Metrics of the VR application running the adaptively streamed version of the Map from the summer on the Standalone VR headset.

Metric	Value
Session Duration (s)	566
Mean FPS	58.62 ± 12.59
Coefficient of Variation	0.215
25th–75th Percentile (FPS)	47.5–71.7

**Table 4:** Performance comparison of the VR application running on the Unity Editor for the Summer Dataset.

Metric	Hydran00 Method 1	Our Method
Session Duration (s)	525.6	536.7
Mean FPS	97.68 ± 29.71	115.84 ± 14.61
Coefficient of Variation	0.304	0.126
25th–75th Percentile (FPS)	95.3–116.2	116.8–122.7

## Data Availability

The data presented in this study are available on request from the corresponding author.

## Abbreviations

The following abbreviations are used in this manuscript:

VR	Virtual Reality
ROS	Robot Operating System
LOD	Level of Detail
3D	Three-Dimensional
HITL	Human-in-the-Loop
HRI	Human–Robot Interaction
DOF	Degree of Freedom
UGV	Unmanned Ground Vehicle
RTABMap	Real-Time Appearance-Based Mapping
URP	Universal Render Pipeline
TCP	Transmission Control Protocol
RGB	Red, Green, Blue
LiDAR	Light Detection and Ranging
2D	Two Dimensional
QoE	Quality of Experience
API	Application Programming Interface
QoS	Quality of Service
